# Weight changes after total knee arthroplasty in Chinese patients: a matched cohort study regarding predictors and outcomes

**DOI:** 10.1186/s13018-019-1184-z

**Published:** 2019-07-02

**Authors:** Pengfei Zan, Jie J. Yao, Kaiyuan Liu, Dong Yang, Weixu Li, Guodong Li

**Affiliations:** 1 Department of Orthopedic Surgery, Second Affiliated Hospital of Zhejiang University, School of Medicine, 88 Jiefang Road, Hangzhou, 310009 People’s Republic of China; 2 Department of Orthopedic Surgery, The Tenth People’s Hospital Affiliated to Tongji University, 301 Yanchang Rd, Shanghai, 200072 People’s Republic of China; 3 Department of Orthopedic Surgery, University of Texas Southwestern Medical Center, Dallas, TX USA

**Keywords:** Total knee arthroplasty, Body mass index, Weight change, WOMAC score, SF-36 score

## Abstract

**Background:**

The purpose of this study was to compare 2-year BMI changes between patients undergoing simultaneous bilateral total knee arthroplasty (TKA), staged bilateral TKA, and unilateral TKA. We also sought to determine the predictors of weight change and whether clinically meaningful weight changes affected outcomes.

**Patients and methods:**

This retrospective, single-institution study included 202 Chinese patients who received simultaneously bilateral TKA, staged bilateral TKA, or unilateral TKA from 2008 to 2015. There were 49 simultaneous bilateral TKAs, 52 staged bilateral TKAs, and a matched 101 unilateral TKAs.

**Results:**

66.8% (135/202) of patients lost weight after TKA surgery. However, 20.7% (42/202) of patients experienced clinically meaningful weight loss (a BMI decrease of more than 5%). Paired *t* test showed that 2-year BMI was significantly lower than preoperative BMI (*p* < 0.001). Weight loss was significantly different between the surgical strategy (*p* < 0.001). Preoperative BMI and age were predictive of clinically significant weight loss or gain (*p* < 0.05). Multiple linear regression showed that post-operative weight loss was associated with better Western Ontario and McMaster Universities Osteoarthritis Index and SF-36 scores (*p* < 0.001).

**Conclusion:**

Patients after TKA experience weight loss. Age and preoperative BMI predict clinically meaningful weight change. Simultaneous bilateral TKA is associated with higher likelihood of weight loss. Clinically meaningful weight loss experiences better patient-reported outcomes.

## Background

Total knee arthroplasty (TKA) is a successful, safe, and cost-effective procedure for reducing pain and restoring function in patients with end-stage knee osteoarthritis [1–4]. Although TKA is effective in obese patients, obesity is associated with prolonged hospital stay [5, 6], increased costs [6, 7], increased blood loss [8], and an increased risk of complications, including surgical site infection [9–14], deep infection [15], and venous thromboembolism [16, 17]. It has also been estimated that more than half of TKA patients have a body mass index (BMI) > 30 in Western countries [18]. Recently, a workgroup of the American Association of Hip and Knee Surgeons Evidence-Based Committee [19] has published a review regarding the effects of obesity on total joint arthroplasty, documenting the adverse effects of obesity in total joint arthroplasty patients and advising that patients with a high BMI receive additional counseling regarding possible increased risk of weight-related complications.

Although many studies have described the effects of obesity on TKA, BMI changes following primary TKA are more controversial [2, 20–26]. One recent systemic review [27] found that a limited number of studies properly addressed the weight changes following TKA and that additional investigation is necessary. To the best of our knowledge, there is no study investigating the BMI changes among patients when stratified by bilateral simultaneous or staged TKA versus unilateral TKA. Furthermore, the vast majority of studies have focused on Western cohorts. Due to differences in racial composition, culture, dietary habits, and esthetic standards between countries, it is essential to document BMI changes following TKA in a Chinese cohort.

Therefore, we were interested in exploring if Chinese patients lose weight after TKA; the different patterns of BMI changes among simultaneous bilateral, staged bilateral, and unilateral TKA patients; and how postoperative BMI changes affect patient-reported 2-year Western Ontario and McMaster Universities Osteoarthritis Index (WOMAC) and SF-36 scores. We hypothesized that TKA surgery will result in 2-year weight loss after TKA surgery; that simultaneous bilateral, staged bilateral, and unilateral TKA patients will experience similar weight loss; and that TKA patients with a clinically meaningful weight loss would have better patient-reported outcome scores than other patients.

### Patients and methods

This retrospective study was designed using prospectively collected data of 202 patients (303 TKAs) who had their TKAs from January 1, 2008, to April 1, 2015. There were 49 simultaneous bilateral TKAs and 52 staged bilateral TKAs at our institution. These were matched in a 1:1 ratio to unilateral TKAs by age (± 5 years), sex, year of operation, and preoperative BMI (± 1 kg/m^2^). All patients had 2 years or greater follow-up. Patients were excluded if they had a diagnosis of a systemic inflammatory disease. For staged bilateral TKA patients, patients were excluded if the interval between surgeries was more than 1 year. Finally, because we wanted to determine BMI changes and the effect on the clinical outcomes only after the successful primary TKA surgeries, we excluded those TKA patients that required any revision TKA. The study was approved by the Human Research Ethics Committee of our hospital, and all methods were performed in accordance with the relevant guidelines and regulations.

Clinical and demographic data was collected in detail before surgery. Patients’ height and weight at admission, 1 year after surgery, and 2 years after surgery were collected. For patients receiving staged bilateral TKA, we used the admission data from of first TKA for analysis. BMI was calculated using the formula BMI = (weight in kg)/(height in meters)^2^. We also calculated Western Ontario and McMaster Universities Osteoarthritis Index (WOMAC) [28] scores (including WOMAC pain score, WOMAC stiffness score, WOMAC function score) as this score is the most commonly used standardized questionnaire to evaluate the condition of patients’ joints. We also calculated 36-item short form health survey (SF-36) [29] scores. The SF-36 scores were also separated by the Physical Component Summary (PCS) and Mental Component Summary (MCS) scores. The SF-36 is an easily administered quality-of-life measures which can evaluate the patients’ overall physical and mental functional status. Both the WOMAC and SF-36 were collected in detail at admission and two postoperative years.

Based on the World Health Organization’s established BMI classification [30], patients were divided into 6 categories: underweight (< 18.50 kg/m^2^), normal weight (18.50 to 24.99 kg/m^2^), overweight (25.00 to 29.99 kg/m^2^), obese class I (30.00 to 34.99 kg/m^2^), obese class II (35.00 to 39.99 kg/m^2^), and obese class III (≥ 40 kg/m^2^). Within our cohort, 70 patients were normal weight, 110 patients were overweight, 20 patients were obese class I, and 2 patients were obese class II (Table 1). No patients were underweight or obese class III. Weight change was calculated as the difference of BMI at 2 years follow-up measurements compared to preoperative BMI. As defined by the US Food and Drug Administration (FDA) [31], a change in BMI of 5% or more can be considered clinically meaningful. Based on this criteria, our patients were also stratified into 3 groups: weight loss (*a* > 5% decrease in BMI), maintaining a stable BMI (*a* ≤ 5% change in BMI), and weight gain (*a* > 5% increase in BMI). We then analyzed the weight change and its effect on outcome measures within each subgroup.

**Table 1:** Baseline demographics and clinical characteristics of the patient population

Parameters	Simultaneous bilateral TKA (*n* = 49)	Staged bilateral TKA (*n* = 52)	Unilateral TKA (*n* = 101)	All TKA patients (*n* = 202)
Age (years)	68.9 ± 7.9	68.9 ± 7.1	69.8 ± 6.7	69.4 ± 7.1
Male/female	17/32	20/32	37/64	74/1128
Height (m)	1.65 ± 0.07	1.64 ± 0.08	1.65 ± 0.07	1.65 ± 0.07
Weight (kg)	70.98 ± 8.55	70.49 ± 7.25	72.31 ± 8.95	71.52 ± 8.44
BMI (kg/m^2^)	26.15 ± 2.74	26.45 ± 3.43	26.49 ± 3.14	26.40 ± 3.12
BMI category				
Normal weight (18.50–24.99)	17 (34.7%)	21 (40.4%)	32 (31.7%)	70 (34.7%)
Overweight (25.00–29.99)	29 (59.2%)	23 (44.2%)	58 (57.4%)	110 (54.5%)
Obese I (30.00–34.99)	3 (6.1%)	8 (15.4%)	9 (8.9%)	20 (9.9%)
Obese class II (35.00–35.99)	0	0	2 (2.0%)	2 (1.0%)
Charlson-Deyo comorbidity index				
0	28	36	41	105 (52.0%)
1 to 2	21	15	58	94 (46.5%)
≥ 3	0	1	2	3 (1.5%)
Total WOMAC score	56.1 ± 4.5	57.0 ± 3.9	56.0 ± 3.9	56.3 ± 4.0
WOMAC pain score	13.1 ± 2.1	13.6 ± 2.1	12.5 ± 2.5	12.9 ± 2.3
WOMAC stiffness score	3.6 ± 1.3	3.9 ± 1.3	3.1 ± 1.5	3.4 ± 1.4
WOMAC function score	39.3 ± 3.5	39.4 ± 3.3	40.4 ± 3.8	39.9 ± 3.6
SF-36 PCS score	35.0 ± 4.5	35.1 ± 5.3	36.0 ± 4.6	35.5 ± 4.8
SF-36 MCS score	45.6 ± 5.0	45.5 ± 6.2	44.4 ± 3.7	45.0 ± 4.8

*BMI* body mass index, *WOMAC* Western Ontario and McMaster Universities Osteoarthritis Index, *Total WOMAC score* WOMAC pain score + WOMAC stiffness score + WOMAC function score, *SF-36* 36-item short form health survey, *PCS* Physical Component Summary, *MCS* Mental Component Summary

### Statistical methods

Descriptive statistics were performed for patients’ baseline demographic characteristics. All data analysis was performed by using SPSS statistics software (SPSS Inc., Chicago, USA). Categorical variables were presented as absolute numbers and relative frequencies. Paired Student *t* tests were used to compare 2-year BMI versus preoperative BMI. This was done both for all TKA patients and for TKA patients stratified by surgical strategy. Kruskal-Wallis H test and Tukey’s HSD were used to evaluate differences in BMI change between TKA patient surgical strategy groups. Binomial logistic regression was used to identify the potential predictors for weight gain and loss. Multiple linear regression analysis was used to examine the effect of 2-year weight change after surgery on patient-reported outcomes, controlling for the preoperative age, sex, BMI, and Charlson-Deyo comorbidity index. A *p* value less than 0.05 was considered as significant difference.

## Results

### Patient selection and demographic baseline data

**Fig. 1:**
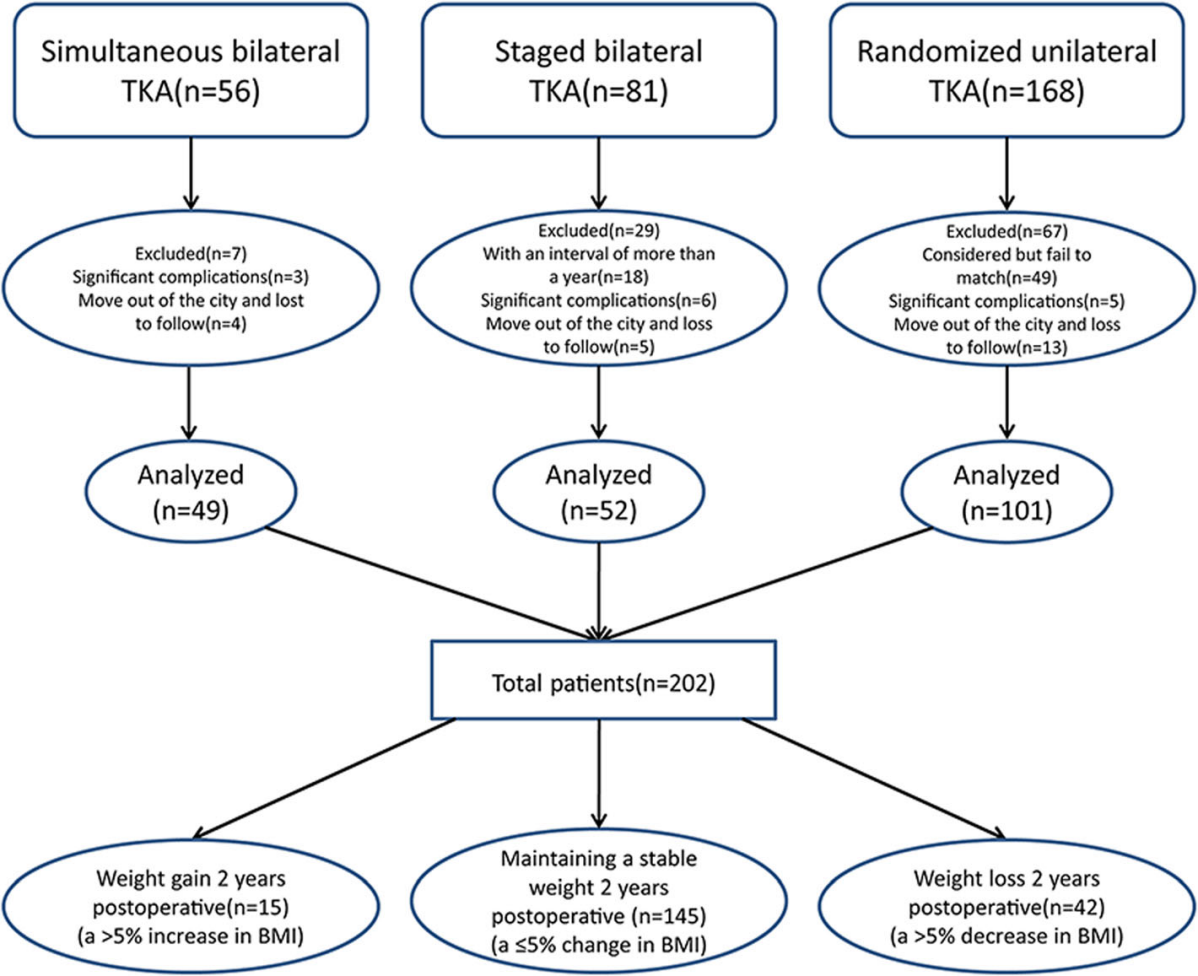
Patient flowchart

A total of 305 primary TKA patients including 56 simultaneous bilateral, 81 staged bilateral, and 168 unilateral TKA patients were identified during the study period. One hundred three patients were excluded from the study leaving a final of 202 patients recruited for this observational study (Fig. 1). The average age was 69.4 ± 7.1 years, and average BMI was 26.4 ± 3.1 kg/m^2^. One hundred twenty-eight patients were female (63.4%).

### Clinical outcome measurements

**Fig. 2:**
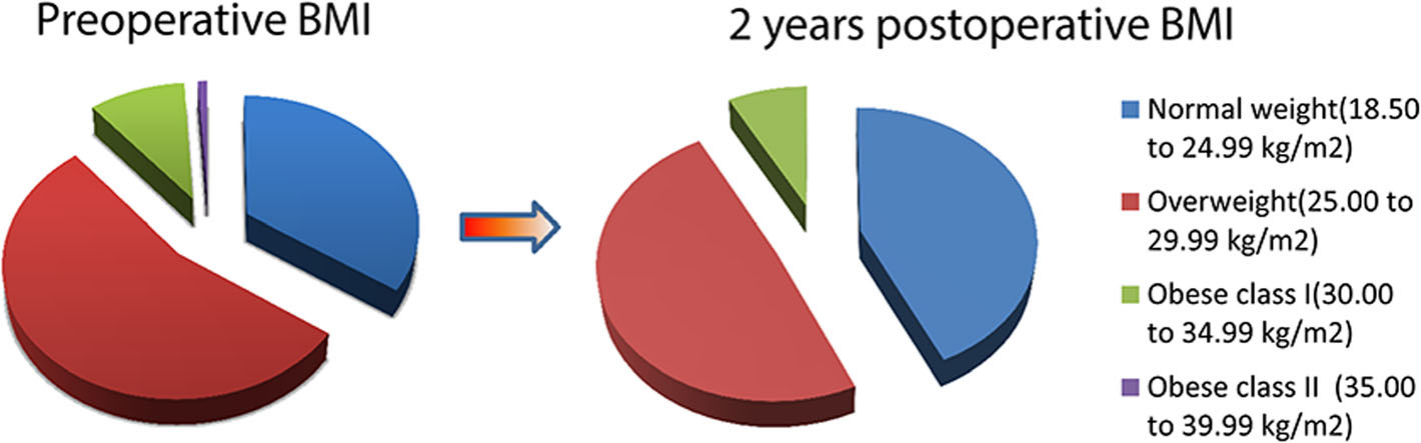
Patients in each BMI class changed with the follow-up time

Overall, 66.8% of patients (135/202) lost some weight after TKA surgery (Table 2). 20.7% (42) TKA patients experienced clinically meaningful weight loss (BMI decrease greater than 5% of preoperative BMI). In the TKA cohort as a whole, mean BMI at 2 years (25.9 kg/m^2^) was significantly lower than preoperative BMI (26.4 kg/m^2^; *p* < 0.001; Table 2). When patients were stratified by surgical strategy, unilateral and simultaneous TKA patients also showed a significantly lower 2-year BMI compared to preoperative BMI (*p* < 0.001). However, staged bilateral TKA patients did not have significantly different preoperative and 2-year BMIs (*p* = 0.712). The patients in each BMI class changed over time (Fig. 2).

**Table 2:** Within-subjects BMI change after TKA surgery

Parameters	Preoperative (BMI)	2 years postoperative (BMI)	Change in BMI, mean (95% CI)	*p* value
All TKA patients (*n* = 202)	26.40 ± 3.12	25.93 ± 2.87	0.470 (0.322 to 0.618)	< 0.001*
Simultaneous bilateral TKA patients (*n* = 49)	26.15 ± 2.74	25.20 ± 2.37	0.950 (0.673 to 1.226)	< 0.001*
Staged bilateral TKA patients (*n* = 52)	26.45 ± 3.43	26.40 ± 3.19	0.046 (− 0.201 to 0.293)	0.712
Unilateral TKA patients (*n* = 101)	26.49 ± 3.14	26.03 ± 2.87	0.456 (0.237 to 0.676)	< 0.001*

*CI* confidence interval

*Significant difference

**Fig. 3:**
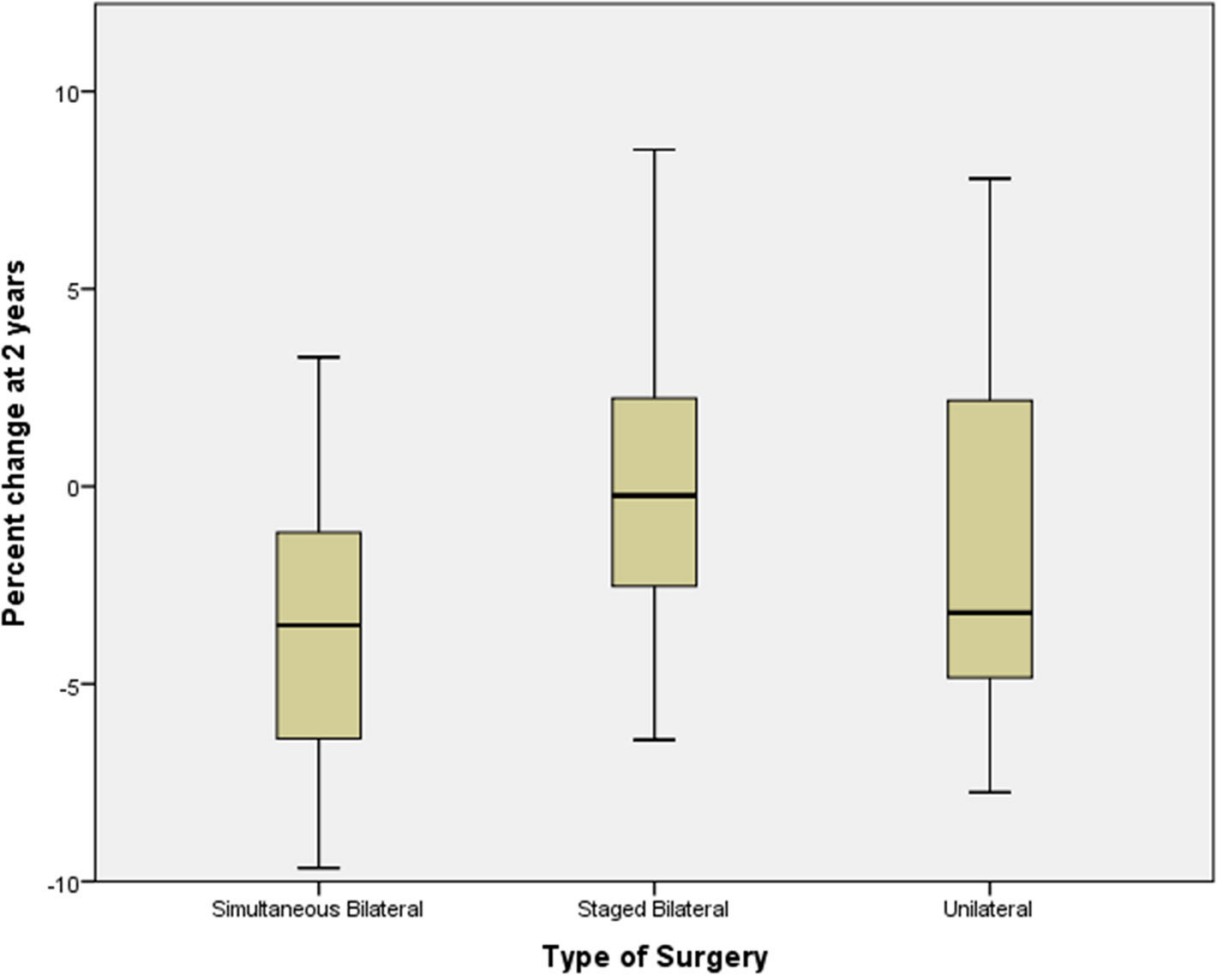
Box plot showed 2-year postoperative BMI stratified by type of surgery

The mean 2-year BMI change (± standard deviation) in all TKA patients was − 0.470 ± 1.067 kg/m^2^. Comparison of the BMI percent change between surgical groups showed that BMI change was significantly different between groups (Fig. 3; *p* < 0.001). A Tukey HSD test revealed simultaneous bilateral TKA patients had a significantly greater percent BMI loss at 2 years compared to the unilateral TKA group (*p* = .047). Percent BMI loss was significantly lower in the staged bilateral TKA group than in the unilateral TKA group or simultaneous TKA groups (*p* = 0.017, *p* < 0.001 respectively).

BMI and age were found to predict clinically meaningful weight loss and weight gain (Table 3). Increased preoperative BMI was associated with increased odds of clinically meaningful weight loss (odds ratio [OR] 1.27; *p* < 0.001). Increased preoperative BMI was associated with decreased odds of clinically meaningful weight gain (OR 0.70; *p* = 0.007). Increased age was associated with increased odds of clinically meaningful weight loss (OR 1.09; *p* = 0.004) and clinically meaningful weight gain (OR 1.09; *p* = 0.048). Sex, preoperative WOMAC score, preoperative SF-36 PCS score, and preoperative SF-36 MCS score were not found to be significant predictors of clinically meaningful weight loss or gain.

**Table 3:** Logistic regression results evaluating the predictors of clinically meaningful weight loss and gain after TKA surgery

Parameters	Weight loss (*n* = 42)^£^	*p* value	Weight gain (*n* = 15) ^£^	*p* value
Preoperative BMI	1.271 (1.123 to 1.440)	< 0.001*	0.698 (0.538 to 0.906)	0.007*
Sex	1.900 (0.794 to 4.548)	0.149	1.161 (0.361 to 3.730)	0.803
Age	1.088 (1.027 to 1.153)	0.004*	1.089 (1.001 to 1.184)	0.048*
Preoperative total WOMAC score	1.066 (0.968 to 1.174)	0.191	0.928 (0.803 to 1.073)	0.313
Preoperative SF-36 PCS score	0.969 (0.893 to 1.050)	0.442	1.088 (0.964 to 1.230)	0.173
Preoperative SF-36 MCS score	1.007 (0.927 to 1.094)	0.869	1.036 (0.926 to 1.159)	0.538

^£^The values are given as the odds ratio, with 95% confidence interval in parentheses

*Significant difference

Clinically meaningful weight loss was associated with lower WOMAC total score (*p* < 0.001; Table 4). Similarly, clinically meaningful weight loss was also associated with lower WOMAC pain, function, and stiffness scores (*p* = 0.001, *p* = 0.002, *p* = 0.015 respectively). Clinically meaningful weight loss was also associated with higher SF-36 PCS and SF-36 MSC scores (*p* < 0.001, *p* = 0.025 respectively). Clinically meaningful weight gain was associated with higher WOMAC total score (*p* < 0.001). Clinically meaningful weight gain was associated with higher WOMAC function and stiffness scores (*p* < 0.001, *p* < 0.001 respectively). WOMAC pain score was not significantly associated with weight gain. Clinically meaningful weight gain was associated with lower SF-36 PCS and SF-36 MCS scores (*p* = 0.001, *p* = 0.003 respectively).

**Table 4:** Multiple linear regression results evaluating the effects of clinically meaningful weight loss and gain on the outcomes after TKA surgery

Parameters	Weight loss (*n* = 42)^¥^	*p* value	Weight gain (*n* = 15) ^¥^	*p* value
WOMAC total score	11.50 ± 2.85	< 0.001*	17.47 ± 1.81	< 0.001*
WOMAC pain score	1.40 ± 0.63	0.001*	2.07 ± 0.80	0.120
WOMAC function score	9.45 ± 2.60	0.002*	13.53 ± 2.17	< 0.001*
WOMAC stiffness score	0.64 ± 0.66	0.015*	1.87 ± 0.92	< 0.001*
SF-36 PCS score	49.38 ± 2.02	< 0.001*	42.4 ± 3.14	0.001*
SF-36 MCS score	50.83 ± 8.19	0.025*	43.80 ± 3.19	0.003*

^¥^Values are given as mean and standard deviation

*Significant difference

## Discussion

To our knowledge, this is the first study investigating BMI trends in a Chinese cohort after primary TKA. Furthermore, previous studies on BMI trends after TKA have not stratified patients by simultaneous bilateral, staged bilateral, and unilateral TKA. In a Chinese cohort, we found that while most patients lost some weight after TKA surgery, a much smaller subset of patients experienced clinically meaningful weight loss. Unilateral TKA and simultaneous TKA patients significantly lost weight at 2 years after surgery. However, staged bilateral TKA patients did not experience a significant BMI change at 2 years after surgery. Clinically meaningful weight change after TKA was also associated with better patient-reported outcome scores.

We demonstrated that most patients (66.8%) lose some weight after TKA surgery, and a lower percentage of patients (20.7%) lose clinically meaningful weight. However, in our cohort, very few patients (7.2%) gained clinically meaningful weight. Our findings are consistent with existing findings. Recently, in a large sample of over 3000 patients, Ast et.al [22] found that 69% of patients demonstrated no significant change at 2 years after surgery. Dowsey et al. [32] found a clinically meaningful weight loss occurred in 12.6% of patients and 21% of patients actually experienced weight gain. Kahn et al. [2] compared TKA patients with osteoarthritis patients who did not received TKA surgery; they found no difference in BMI. However, Zeni and Snyder-Mackler [33] compared TKA patients with a healthy control group and found that most patients gained weight 2 years postoperatively. Chang et al. [34] conducted a retrospective review of Korean cohorts and found that 70.2% of patients maintained a stable BMI and 12.6% of patients had a clinically meaningful weight loss. A recent systematic review [27] found that existing studies were very low quality and failed to properly address the weight changes following TKA. They were unable to conclude whether weight increases, decreases, or remains the same after TKA.

We also demonstrated that surgery strategy affected BMI change after TKA surgery. Compared with unilateral TKA, simultaneous bilateral TKA patients had significant weight loss. However, staged bilateral TKA surgery was less likely to benefit patients’ weight decreasing in a 2-year follow-up. The BMI change in simultaneous bilateral TKA patients was significantly greater than the BMI change in unilateral TKA patients. This may be related to increased mobility following simultaneous bilateral TKA. Unilateral TKA patients may have contralateral osteoarthritis. This osteoarthritis may not be significant to require TKA, but may still limit mobility and function. It is unclear why staged bilateral TKA surgery patients did not show a significant change in BMI at 2 years. The interval period between operations may contribute to weight trends as patients may be less mobile in between TKA surgeries.

We also wanted to investigate significant predictors that might contribute to BMI change after TKA surgery. We found that preoperative BMI and age predicted clinically meaningful weight loss and weight gain. Increased preoperative BMI was associated with higher likelihood to lose a clinically meaningful weight, and increased age was less likely associated with clinically meaningful weight loss and clinically meaningful weight gain. However, sex, preoperative WOMAC score, preoperative SF-36 PCS score, and preoperative SF-36 MCS score were not found to be significant predictors of clinically meaningful weight loss or gain. Chang et al. [34] found patients with a lower preoperative BMI were associated with more weight gain. Zeni and Snyder-Mackler [33] tried to determine some indicators for weight gain, but they did not find any significant relationships between weight change and educational level, marital status, income level, or activity level prior to surgery. In Ast et al.’s [22] study, they demonstrated that preoperative obesity and female sex were significant predictors of weight loss.

Finally, we examined whether BMI change after surgery was associated with patient-reported outcomes. We found that patients with clinically meaningful weight had significantly better patient-reported outcomes. Patients with clinically meaningful weight gain had inferior outcomes. Our findings are in accordance with some previous studies. Mackie et al. [26] demonstrated that weight gain over 10% had a negative impact on the SF-36 pain and functional scores. Ast et al. [22] found that weight gain after TKA surgery was associated with inferior scores on the SF-36 PCS score. However, weight loss was not associated with better outcome scores despite seeing better outcome scores in total hip arthroplasty patients with weight loss. Additionally, many studies have demonstrated that weight gain after TKA was associated with higher incidence of complications and shorter time to revision [30, 35, 36]. Given the evidence presented above, orthopedic surgeons should counsel patients to avoid weight gain after TKA to reduce the adverse influences of weight gain on the clinical outcomes.

Our findings need to be interpreted in light of some potential limitations. Firstly, our sample size is small compared to some previous studies. However, we were most interested in the bilateral TKAs, limiting our sample size as this is not common practice at our hospital. Secondly, patient-reported WOMAC and SF-36 scores are subjective surveys which do suffer from bias. Furthermore, some patients may misinterpret questions on surveys; however, an independent nurse provided interpretation and instruction on the surveys for all the patients. Thirdly, our cohorts were comprised completely of Chinese individuals, and the mean BMI was notably lower than has been reported in many Western cohorts. While this is one unique aspect of our cohort, our findings may not be generalizable to groups of different racial proportions, cultures, diet habits, and esthetic standards. Though these potential limitations exist, our present study provides some evidence regarding the BMI changes, predictors, and outcomes in simultaneous bilateral, staged bilateral, and unilateral TKA Chinese patients.

## Conclusion

In a Chinese cohort, most patients lost weight after TKA surgery; however, fewer patients lost clinically meaningful weight. Simultaneous bilateral and unilateral TKA patients had significantly decreased BMI at 2 years. Clinically meaningful weight loss after TKA surgery was associated with more satisfied patient-reported outcomes. TKA patients should be advised regarding that weight loss after TKA can improve outcomes.

## Abbreviations

BMI: Body mass index
MCS: Mental component summary
PCS: Physical component summary
SF-36: 36-item short form health survey
TKA: Total knee arthroplasty
WOMAC: Western Ontario and McMaster Universities Osteoarthritis Index
